# Astrocytes: a star emerges in the control of reproductive hormones

**DOI:** 10.1172/JCI182669

**Published:** 2024-08-01

**Authors:** Ali Abbara, Waljit S. Dhillo

**Affiliations:** 1 Section of Endocrinology and Investigative Medicine, Division of Diabetes, Endocrinology and Metabolism, Imperial College London, London, United Kingdom.; 2 Department of Endocrinology, Imperial College Healthcare NHS Trust, London, United Kingdom.

## Abstract

Kisspeptin is an essential neuropeptide sitting at the apex of the hypothalamo-pituitary-gonadal (HPG) endocrine axis to regulate gonadotropin-releasing hormone (GnRH) neurons and downstream reproductive hormones. Kisspeptin neurons integrate feedback from sex steroids facilitating regulation of the menstrual cycle and mediate the effects of metabolic stressors on the reproductive axis. In this issue of the *JCI*, Torres and colleagues describe another pathway for kisspeptin signaling in astrocytes to influence GnRH neuronal output. Astrocytes had kisspeptin receptors that activated canonical intracellular signaling pathways to constrain the magnitude of kisspeptin-induced GnRH neuronal stimulation. Additionally, the appositions between kisspeptin and GnRH neurons were dynamic during the ovarian cycle, with astrocyte kisspeptin signaling proposed as a putative modulator of this neuroplasticity. Importantly, astrocyte kisspeptin signaling also mediated susceptibility to metabolic stressors and the development of obesity-induced hypogonadism, underscoring the physiological and pathological importance of this pathway and revealing the importance of nonneuronal signaling in reproductive health.

## Kisspeptin regulates GnRH neurons in the hypothalamus

Kisspeptin is essential for reproductive health; inactivating variants in genes encoding for kisspeptin or its receptor result in hypogonadotropic hypogonadism and a failure to initiate pubertal development (1, 2). Conversely, excess kisspeptin signaling results in early activation of the reproductive endocrine axis and precocious puberty (3). Following a plethora of further research across several species, kisspeptin is now recognized to be a key regulator of reproductive hormones and controls the secretion of gonadotropin releasing hormone (GnRH) from dedicated neurons in the hypothalamus (4, 5).

Kisspeptin resides in two major neuronal populations in the hypothalamus; first, in the arcuate nucleus (ARC), where it regulates the physiological pulsatile secretion of GnRH, and second, in the preoptic area (POA), which includes the anteroventral periventricular nucleus (AVPV) in animal models, where it is responsible for the midcycle ovulatory luteinizing hormone (LH) surge (6). Kisspeptin neurons in the ARC coexpress neurokinin B (NKB) and dynorphin and are commonly referred to as KNDy neurons (7, 8). These neuropeptides act in concert in an auto/paracrine manner to regulate the activity of KNDy neurons, with NKB being a positive regulator and dynorphin a negative regulator of their activity. The output of these KNDy neurons is kisspeptin, which acts on GnRH neuronal dendrons through nonsynaptic appositions via short-distance volume transmission (9). Thus, ARC kisspeptin neurons are believed to be the ‘GnRH pulse generator’ playing a critical role in the physiological pulsatile secretion of GnRH and downstream reproductive hormone secretion.

Kisspeptin neurons are a key conduit for the integration of peripheral signals into the reproductive axis, including sex steroids and metabolic markers. Kisspeptin neurons mediate feedback loops needed for the functional operation of the menstrual cycle, with estradiol acting via ARC kisspeptin neurons to cause negative feedback on pulsatile GnRH secretion during the follicular phase of the menstrual cycle. In contrast, higher levels of estradiol found towards the end of the follicular phase act on kisspeptin neurons in the POA to induce positive feedback and the mid-cycle LH surge responsible for ovulation (6). As the LH surge is of relevance only in females, AVPV kisspeptin neurons are far more prominent in females than males. Indeed, kisspeptin is generally more critical to female reproductive function, with less hypothalamic kisspeptin expression being required in males to maintain fertility (8).

Overall, kisspeptin neurons play a pivotal role in the regulation of GnRH neurons and the reproductive axis; however, the relevance of kisspeptin signaling in other nonneuronal brain cells is far less studied. In this issue of the *JCI*, Torres and colleagues provide the first description of kisspeptin modulating the activity of nonneuronal cells in the brain, akin to the seminal appreciation of the functional importance of noncoding regions of the genome (10).

## Kisspeptin signaling in nonneuronal brain cells

The investigators originally applied label-free proteomics in the POA of the hypothalamus (where GnRH neurons are situated) to identify protein targets of kisspeptin signaling (10). Different protein clusters were identified following kisspeptin stimulation, but the investigators were intrigued by the increase in glial fibrillary acidic protein (GFAP) and vimentin, and the decrease in Amyloid Precursor Protein (APP) and Metallothionein 3 (MT3) levels (10). These markers are present in astrocytes, suggesting that kisspeptin stimulation could affect these nonneuronal brain cells.

Astrocytes are the most common subtype of glial cell and provide structural support to neurons to ensure that synapses are apposite and aligned. They play an important role in neuronal sustenance, hosting energy sources such as glycogen and acting as a conduit for the passage of nutrients from blood vessels. Additionally, astrocytes have an important role in maintaining the blood-brain barrier (BBB) and regulating regional blood flow. Notably, astrocytes can also influence neuronal signaling via the uptake and recycling of excess neurotransmitters from synapses back to neurons as well as by releasing gliotransmitters, which are substances released from astrocytes such as glutamate, adenosine, or adenosine triphosphate that modulate neuronal synaptic activity and plasticity. In particular, astrocytes envelop GnRH neurons and can modulate their activity; for example, astrocytes can release prostaglandin E_2_ (PGE_2_) to stimulate GnRH neuronal activity. Overall, astrocytes are important supportive neural cells but could also play a role in modulating neuronal signaling.

Torres et al. provided further evidence of a direct effect of kisspeptin on astrocytes by demonstrating the presence of the kisspeptin receptor (but not kisspeptin) in murine and human astrocytes (10). Typically, activation of the G-protein–coupled kisspeptin receptor on GnRH neurons triggers Gα_q/11_ and activation of Phospholipase C (PLC), leading to inositol triphosphate (IP3) and diacylglycerol (DAG), which activate Protein Kinase C (PKC), resulting in stimulation of the mitogen-activated protein kinase (MAPK) signaling cascade and ERK1/2 (11, 12). Torres and colleagues demonstrated that kisspeptin, likewise, induced phosphorylation of ERK1/2 in rodent astrocyte culture, consistent with kisspeptin activating its canonical signaling pathway in these cells (10).

Interestingly, Torres and investigators noted regional variation in kisspeptin’s induction of signaling in astrocytes, with colocalization of astrocyte markers and the kisspeptin receptor being greater in GnRH- and kisspeptin-rich areas, but less in cortical areas from which astrocytes did not respond to kisspeptin stimulation (10). Indeed, close appositions between astrocytes and kisspeptin neurons were demonstrated, especially in key hypothalamic areas such as the ARC and anteroventral periventricular nucleus (AVPV), providing a likely source of kisspeptin to act on astrocyte kisspeptin receptors and consistent with a putative modulatory action of astrocytes on the response to kisspeptin in GnRH neurons. Appositions between kisspeptin and GnRH neurons were dynamic during the ovarian cycle, and notably more than one third of differentially expressed proteins in response to kisspeptin stimulation were markers of synaptic plasticity (10). Overall, these data suggest that astrocytes could play a role in modulating neuroplasticity in the interactions between kisspeptin and GnRH neurons.

**Figure 1:**
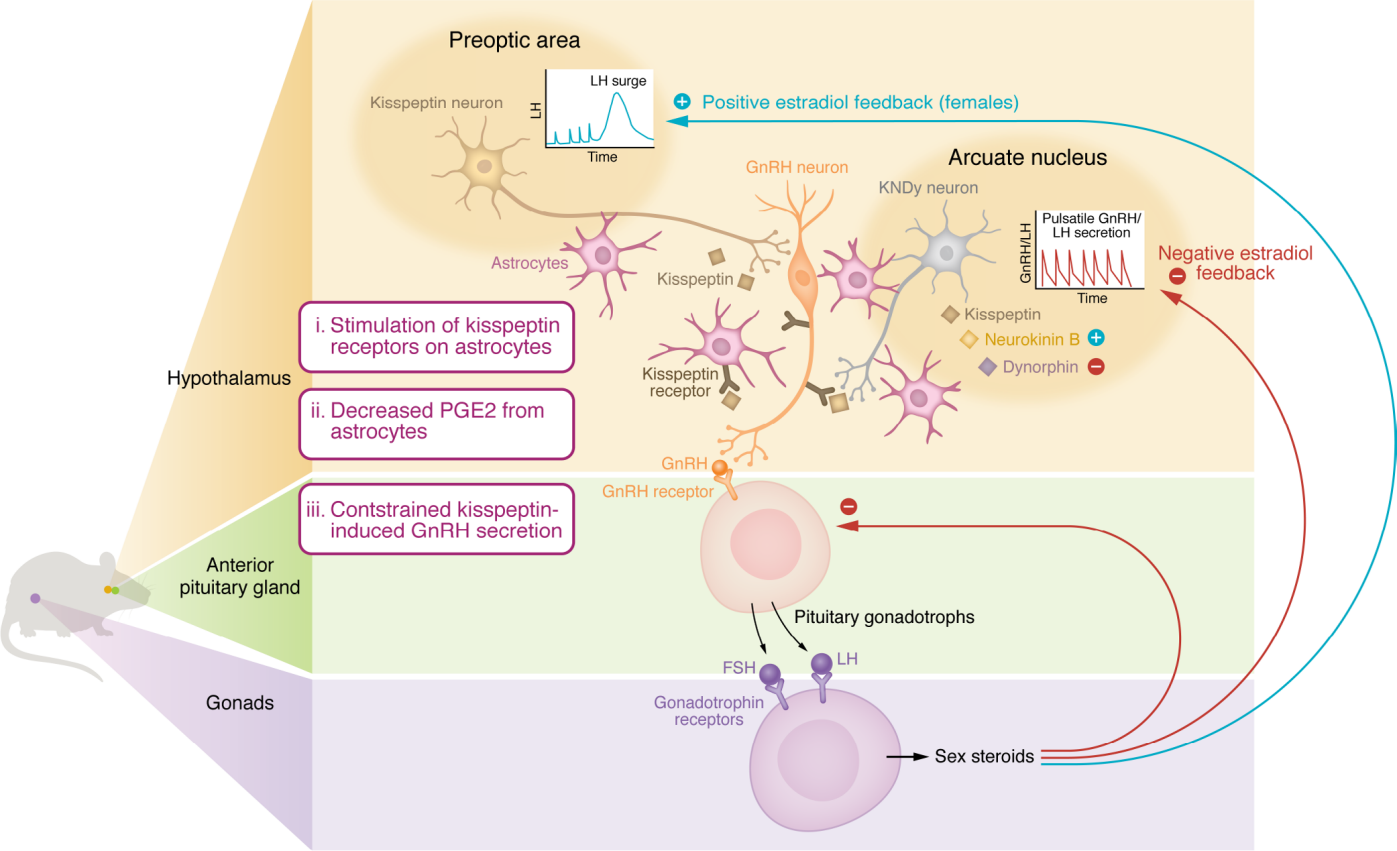
Kisspeptin signaling in astrocytes. Kisspeptin stimulation of kisspeptin receptors on astrocytes decreases their production of prostaglandin E2 (PGE2). PGE2 is a stimulatory input onto GnRH neurons. Consequently, the kisspeptin-astrocyte signaling pathway constrains kisspeptin-induced stimulation of GnRH neurons. Kisspeptin neurons in the arcuate nucleus of the hypothalamus are responsible for pulsatile GnRH/LH secretion and respond to negative feedback from sex steroids. Kisspeptin neurons in the preoptic nucleus of the hypothalamus respond to positive feedback from higher levels of sex steroids to induce the midcycle ovulatory LH surge in females. FSH, Follicle Stimulating Hormone; LH, Luteinizing Hormone; GnRH, Gonadotropin Releasing Hormone; KNDy, Kisspeptin Neurokinin B, Dynorphin.

The researchers further investigated the role of kisspeptin in astrocytes by creating a G-KiR-KO mouse model, whereby the kisspeptin receptor was ablated from GFAP-expressing cells (a marker present in astrocytes) (10). Although there was no impact on pubertal timing in male or female mice in G-KiR-KO mice, the LH response to kisspeptin was increased, particularly in female mice (10). This observation is consistent with kisspeptin-astrocyte signaling having a constraining effect on the response of GnRH neurons to kisspeptin. Congenital ablation of the kisspeptin receptor from astrocytes resulted in upregulated PGE_2_, which is a major stimulatory signal for GnRH neurons (10). Hence, kisspeptin’s action in astrocytes could plausibly restrain PGE_2_ to curb GnRH neuronal stimulation (Figure 1).

G-KiR-KO mice had more appositions between astrocytes and kisspeptin neurons but lower basal LH secretion, albeit with a trend towards more LH pulses. If kisspeptin’s action in astrocytes was suppressive, increased, rather than reduced, LH levels would be expected in G-KiR-KO mice (10). Hence, the investigators suggest that partial desensitization of kisspeptin receptors could explain this incongruity. Nevertheless, as no major reproductive phenotype was observed in G-KiR-KO mice, kisspeptin’s action in astrocytes is likely modulatory and involved in fine tuning rather than fundamental, as it is in GnRH neurons, as ablation of the kisspeptin receptor from GnRH neurons results in absent GnRH secretion. Although neuroprogesterone synthesis in astrocytes has been suggested to mediate estradiol-induced positive feedback, G-KiR-KO mice also did not have any abnormality of the ovulatory LH surge (10).

Obesity suppresses activity of ARC kisspeptin neurons leading to obesity-induced hypogonadism (13). In obese male mice, kisspeptin neurons have fewer glutamate and melanocortin 4 receptors (14). Although a high-fat diet (HFD) advanced initiation of puberty in female mice (albeit not in male mice), Torres and colleagues did not find this effect in G-KiR-KO mice (10). Notably, while G-KiR-KO mice had preserved reproductive function under normal feeding conditions, LH pulses were disturbed under a HFD with lengthening of estrus cycles (10). Thus, kisspeptin signaling in astrocytes might help protect against the deleterious effects of a HFD on the reproductive axis. Interestingly, G-KiR-KO mice had improved glucose homeostasis that was not mediated by changes in insulin sensitivity or any differences in bodyweight, but the mechanism for this finding has yet to be fully elucidated.

## Conclusions and implications

The data presented in Torres et al. (10) highlight the potential role of kisspeptin signaling in astrocytes in regulating GnRH neuronal activity. While the complexity of neuronal pathways in the brain was already evident, these data suggest that our existing understanding may in fact represent an oversimplification of the true intricacies involved. Even the advance of brain organoids may struggle to fully recapitulate the complexities and dynamic context-specific plasticity present in vivo (15). Indeed, in addition to these findings, emerging data propose that kisspeptin could also have actions in other nonneuronal cells such as oligodendrocytes, which maintain myelin sheaths around neurons, and microglia, which are the primary immune cells in the brain. For example, signaling in nonneuronal cells could play a more prominent role in mediating estradiol feedback than is currently recognized (16). Additionally, kisspeptin’s action via microglia has been proposed to modulate the effect of senescence on the reproductive axis (17). Animal models of polycystic ovary syndrome (PCOS) are typically derived by exposure to androgens at various stages of development. Microglia are proposed to play an important role in establishing aberrant neuronal circuitry in response to abnormal sex steroid exposure (18), and, thus, potentially also in the increased kisspeptin neuronal activity key to the pathogenesis of PCOS (19). Moreover, there is increasing recognition of potential roles of GnRH and kisspeptin beyond their more archetypal functions in the reproductive axis, including in Parkinson’s disease (20), cognition (21), and mood (22). Thus, it is likely that further uncovering the roles of nonneuronal cells, both in the reproductive axis and beyond, will continue to be an important focus of study.

Overall, these data represent an important step forward in our recognition of the impact of kisspeptin signaling within nonneuronal cells in the brain on the function of established neuronal pathways, yielding both physiological and pathophysiological importance.

## Version 1 08/01/2024

Electronic publication
